# Oxidative Stress, Metabolic Impairment and Neuroinflammation are Associated With Target Organ Damage in SHRSP

**DOI:** 10.33549/physiolres.935659

**Published:** 2025-10-01

**Authors:** Silvie HOJNÁ, Lucia MRÁZIKOVÁ, Anna SHÁNĚLOVÁ, Helena PELANTOVÁ, Augusto MONTEZANO, Rhian M. TOUYZ, Lenka MALETÍNSKÁ, Jaroslav KUNEŠ

**Affiliations:** 1 Institute of Physiology, Czech Academy of Sciences, Prague, Czech Republic; 2 Institute of Organic Chemistry and Biochemistry, Czech Academy of Sciences, Prague, Czech Republic; 3 First Faculty of Medicine, Charles University, Prague, Czech Republic; 4 Institute of Microbiology, Czech Academy of Sciences, Prague, Czech Republic; 5 Research Institute of the McGill University Health Centre, Montreal, Canada; 6 Department of Medicine and Department of Family Medicine, McGill University, Montreal, Canada

**Keywords:** SHRSP, Neuroinflammation, Oxidative stress, Metabolomics

## Abstract

Stroke-prone spontaneously hypertensive rats (SHRSP) are widely used as a model to study cerebral small vessel disease (CSVD) and its association with chronic hypertension. This study investigated the relationship between metabolic, cardiovascular, and neuronal comorbidities in 32-week-old SHRSP rats versus Wistar-Kyoto (WKY) controls, with a focus on oxidative stress, inflammation, and metabolic alterations. Despite hypertension and cardiac and renal hypertrophy, no significant cerebral vascular changes or microbleeds and no cerebral edema were detected in SHRSP. NMR-based urinary metabolomics revealed reduced gut microbiome-derived metabolites, such as p-cresylglucuronide, hippurate, and phenylacetylglycine, alongside increases in methylamine and dimethylamine. These findings reflect gut dysbiosis and altered microbial composition in hypertensive conditions. Elevated markers of oxidative stress, including thiobarbituric acid-reactive substances, and increased expression of NADPH oxidase (NOX) 2 and NOX4 in peripheral tissues suggested oxidative damage in SHRSP rats. Astrocytic hyperreactivity, indicated by increased expression of glial fibrillary acidic protein in brain cortex and hippocampus, was suggestive of neuroinflammatory responses. Our findings highlight complex interplay between hypertension, metabolism, and neuroinflammation while underscoring the variability in SHRSP models.

## Introduction

Stroke-prone spontaneously hypertensive rats (SHRSP) were developed by Okamoto *et al.* in 1974 as an experimental model for studying chronic hypertension and hypertensive-related changes in the brain [1]. These rats naturally develop cerebral edema without any artificial intervention and serve as a suitable model for human cerebral small vessel disease (CSVD). CSVD affects the capillary bed, arterioles and small arteries in the brain [2]. Magnetic resonance imaging studies in humans have shown that CSVD is associated with progressive stages of cognitive decline and stroke-like symptoms [3,4].

High blood pressure is one of the major modifiable risk factors for cerebrovascular disorders, including CSVD. Pathological changes caused by hypertension are closely linked to brain dysfunction and cognitive decline. It is reasonable to assume that consistent control of blood pressure could optimize brain perfusion and potentially reduce the risk of vascular dementia [3,5]. In hypertensive encephalopathy, persistent vasoconstriction of cerebral capillaries results in reduced diameter and loss of elasticity. Degeneration of elastin fibers and collagen deposition further alter the properties of the capillary bed in brain. Oxidative stress and inflammation, driven by pro-inflammatory cytokines like tumor necrosis factor alpha (TNFα), contribute to vascular calcification.

Although advancements in antihypertensive therapy have significantly reduced stroke incidence, their impact on cognitive function has been inconsistent [5,6]. Stroke in SHRSP also causes neuronal death, with astrocytes showing diminished support for neurons. Hypoxia, partly caused by oxidative stress, plays a key role in this neuronal death [7].

Several recent studies highlight SHRSP as a unique animal model for studying spontaneously developing inflammation and endothelial dysfunction, which contribute to early brain injury [8–10]. On the other hand, many studies on SHRSP show variable results, as the strain’s characteristics can differ depending on the source. Brain changes also occur at varying ages, differing from the original animals developed by Okamoto *et al.* [1].

The purpose of this study was to clearly investigate how or if SHRSP rats originating from Charles River (Wilmington, MA, USA) may differ from the original ones developed by Okamoto *et al.* [1]. We chose the age of 32 weeks, at which we predicted the occurrence of pathologies characteristic of the SHRSP strain. Therefore, we studied SHRSP rats and their controls, Wistar-Kyoto rats (WKY), and examined the relationship between metabolic, cardiovascular, and brain comorbidities, comparing our results with previously published studies. Additionally, we employed NMR-based metabolomics in urine to identify metabolites associated with these comorbidities.

## Materials and Methods

### Animals

Inbred SHRSP and WKY male rats as controls were purchased from Charles River (Wilmington, MA, USA) at the age of 4 weeks. The animals were placed in an animal facility with 12:12-h light-dark cycle (lights on at 4:00 AM) and provided *ad libitum* with tap water and standard rodent diet Ssniff® R/M-H (Ssniff Spezialdiäten GmbH, Soest, Germany) containing 33 % protein, 9 % fat and 58 % carbohydrates.

All the procedures and experimental protocols were approved by the Ethical Committee of the Institute of Physiology, Czech Academy of Sciences, and conformed to the European Convention on Animal Protection and Guidelines for Research Animal Use by the Act of the Czech Republic Nr. 246/1992.

### The experimental design

A total of 8 SHRSP and 8 WKY control animals were housed two per cage and their BW was measured weekly. The experiment was terminated at 32 weeks of age, a stage at which CSVD, cortical microbleeds, and strokes are expected to have manifested in SHRSP rats. The rats were fasted overnight in the metabolic cages to collect urine for metabolomics and for functional kidney analysis. Plasma samples were collected from the tail veins to determine biochemical parameters. Rectal body temperature was measured, and OGTT was performed. Then, the animals were deeply anesthetized with pentobarbital (50 mg/kg of BW, IP; Merck, Darmstadt, Germany) and transcardially perfused with 0.9 % ice-cold saline supplemented with heparin (10 U/ml; Zentiva, Prague, Czech Republic). Tissue samples, heart, kidney and brain were dissected, weighted and except of right kidney and brain tissue they were snap frozen in a liquid nitrogen and stored at −80 °C for later analysis. The left brain hemispheres were fixed in 4 % paraformaldehyde (PFA) in phosphate buffer saline (pH 7.4) and used for immunohistochemistry (IHC) and histology, while the second brain hemispheres were used for isolation of the brain cortex and hippocampus and then frozen in a dry ice and stored at −80 °C for Western blot analysis.

### Oral glucose tolerance test

OGTT was conducted following an overnight fasting. Initially, at time point 0, blood samples were collected from the tail veins for basal glucose and insulin levels determination. Subsequently, a glucose solution was administered orally *via* gavage, at a dose of 2 g/kg of BW. Blood glucose concentration was determined in whole blood at intervals of 15, 30, 60, 120 and 180 min using a glucometer (Arkray, Tokyo, Japan). At the same time, the plasma samples for determination of insulin level were taken at intervals of 30, 60 and 120 min. The AUC for glycemia and insulinemia were calculated, representing the change in glucose resp. insulin levels over the specified time intervals.

### Determination of biochemical parameters

Colorimetric assays were used to determine plasma levels of urea, CHOL, TG (Erba Lachema, Brno, Czech Republic) and FFA (Roche, Mannheim, Germany). The plasma insulin concentration was determined using a radioimmunoassay kit (Merck, Darmstadt, Germany). An ELISA immunoassay kit was used to determine plasma levels of leptin (Merck, Darmstadt, Germany) and MCP-1 (Invitrogen, Vienna, Austria). All measurements were performed according to the manufacturer’s instructions. The concentration of CRP in fasting plasma was determined using a Mouse CRP ELISA kit (Thermo Scientific, Frederick, MD, USA), the plasma level of TNFα using TNF alpha rat uncoated ELISA kit and IL-6 using Rat IL-6 uncoated ELISA kit (both Thermo Fisher Scientific, Inc., L Waltham, MA, USA).

### NMR-based metabolomics in urine

Urine samples for NMR metabolomics, collected overnight in metabolic cages, were prepared and measured as described in our previous paper [11]. A 200 μl aliquots were mixed with 340 μl H_2_O and 60 μl phosphate buffer (1.5 M KH_2_PO_4_ in D_2_O, pH 7.4) and transferred to a 5-mm NMR tube. NMR data were acquired on a 600 MHz Bruker Avance III spectrometer (Bruker BioSpin, Rheinstetten, Germany) equipped with a 5 mm TCI cryogenic probe head. All the experiments were performed and processed using Topspin 3.5 software. Proton spectra were measured using a Carr-Purcell-Meiboom-Gill pulse sequence to suppress broad background signals of urinary proteins. The metabolite identification was confirmed using TOCSY and HSQC experiments performed for the selected sample. The parameters of all experiments are described in detail in our previous paper [11].

After excluding the regions with water and urea signals, the spectra were normalized in ProcFlow software online version 1.4 (www.nmrprocflow.org) using the constant sum normalization method. For the untargeted analysis spectra in the range 9.5–0.8 ppm were equidistantly binned (bin width=0.04 ppm) and Pareto scaled. Multivariate statistical analysis was carried out using MetaboAnalyst 6.0 software. The principal component analysis (PCA) was applied to assess trends in the sample grouping and detect possible outliers. The partial least-squares-discriminant analysis (PLS-DA) was performed to build statistical model, validated using leave-one-out cross-validation. The results of the PLS-DA model aggregated in variable importance in projection scores were used for the identification of the bins contributing the most to the group differentiation.

Subsequent targeted analysis was based on the normalized intensities of all well-resolved non-overlapping signals or parts of multiplets. Individual metabolites were identified using Chenomx NMR Suite software (Chenomx Inc., Edmonton, AB, Canada), by comparison with the Human Metabolome Database (www.hmdb.ca) and previously published data.

### Western blot analysis

Frozen tissues (heart, kidney, brain cortex and hippocampus) were processed, and immunoblotting was performed as previously described [12]. The specific primary antibodies were used: NOX2, NOX4, HIF1α and AQP4 were purchased from Abcam (Cambridge, UK) and DCX from Cell Signaling Technology (Beverly, MA, USA). All of them were used in 1:1000 dilution in Tris-buffered saline with Tween-20 and 3 % of bovine serum albumin. Protein levels were normalized to glyceraldehyde 3-phosphate dehydrogenase (GAPDH; Abcam, Cambridge, UK, dilution 1:5000), which served as a housekeeping control. The following secondary antibody was used: StarBright Blue 700 Goat Anti-Rabbit IgG (Bio Rad, Hercules, CA, USA) in 1:2500 dilution.

### Measurement of lipid peroxidation

Lipid peroxidation in the kidney samples was monitored by measuring TBARS formation [13]. The frozen thawed 10 % homogenates in a lysis buffer (20 mM KH_2_PO_4_, 1 mM EGTA, aprotinin, leupeptin and pepstatin in a dilution 1:1000 and 1 mM PMSF) were incubated with thiobarbituric and acetic acid at 95 °C for 45 min. Absorbance was measured at 535 nm and the results were expressed as nmol of MDA/mg of protein.

### Kidney histological evaluation

The kidneys for histological analysis were fixed in 4 % PFA, dehydrated and embedded in paraffin. The sections stained with hematoxylin-eosin and periodic acid Schiff reaction were examined and evaluated in a blind-test fashion. Renal damage evaluated as glomerulosclerosis index (GSI) and tubulointerstitial injury (TII) as well as morphological changes like mean glomerular area (MGA) were examined as described previously [14,15] using the Nikon NIS-Elements AR 3.1 morphometric program (Nikon, Tokyo, Japan).

### Immunohistochemistry and brain histology

PFA-fixed brain hemispheres were processed as previously described [16] with minor modifications. The 50 μm coronal sections were incubated in anti-DCX (Cell Signaling Technology, Beverly, MA, USA, dilution 1:600), anti-GFAP (Invitrogen/Thermo Fisher Scientific, dilution 1:200), and anti-Col IV (Abcam, Cambridge, GB, dilution 1:400) primary rabbit antibodies overnight at 4 °C. A biotinylated goat anti-rabbit secondary antibody (Vectastain ABC Kit, Vector Laboratories, Burlingame, CA, USA) was used for the incubation of free-floating sections at room temperature for 90 min to perform chromogenic IHC. Quantitative assessment of immunostained slices was performed under IX83 P1ZF Olympus Microscope (Olympus Corporation, Tokyo, Japan) equipped with a DP74 camera with bright field using OLYMPUS CellSens Dimension software. All IHC-stained sections were analyzed using ImageJ software.

The same free-floating brain sections as used for IHC were also used for histology. Slices were stained in hematoxylin and eosin, as describe in previous papers [17,18].

### Statistical analysis

The data are presented as the means ± S.E.M.s. Statistical analysis was performed using two-way ANOVA, followed by Sidak’s multiple comparisons test or by unpaired *t*-test, as indicated in the figure legends and tables, with GraphPad Prism software (GraphPad Software, San Diego, CA, USA). The differences were considered significant at p<0.05.

## Results

### Morphometric and metabolic parameters

**Fig. 1:**
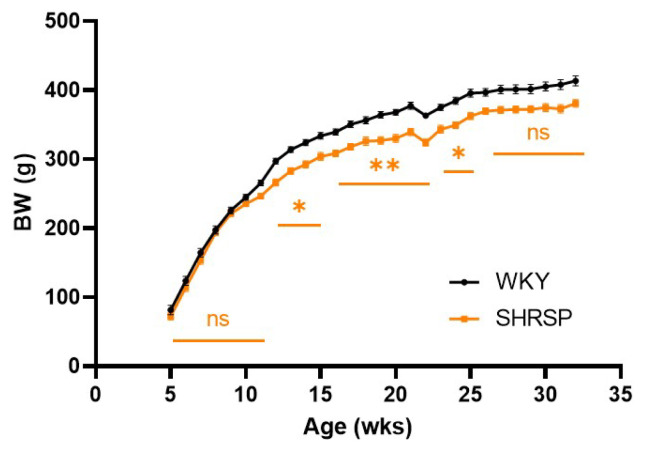
Data are presented as means ± S.E.M. Statistical analysis was performed by *t*-test. Significance is ** p<0.01, *** p<0.001, **** p<0.0001 SHRSP vs. WKY (n=7–8), BW, body weight; FFA, free fatty acids; TG, triglycerides; CHOL, cholesterol.

Despite the same genetic background, SHRSP rats had significantly lower body weight (BW) compared with control WKY (Fig. 1). The final BW is presented in Table 1. Body temperature and several measured metabolic parameters in plasma (free fatty acids-FFA, and triglycerides-TG) were not different between SHRSP and WKY rats (Table 1). On the other hand, SHRSP rats showed significantly lower levels of total cholesterol and leptin in plasma compared to WKY rats. SHRSP rats had significantly increased relative weight of the heart and kidney in comparison with controls. We did not measure blood pressure in this study but previous studies reported significantly elevated BP in SHRSP [19–21].

### No mark of peripheral inflammation

**Fig. 2:**
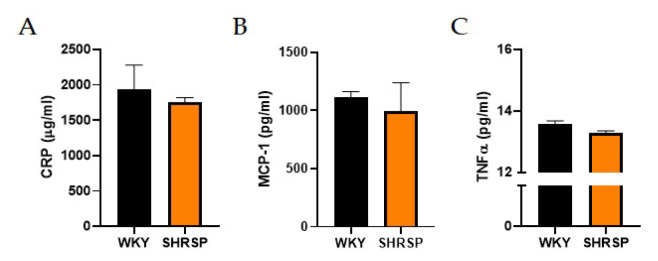
Markers of peripheral inflammation. (**A**) Plasma concentration of C-reactive protein (CRP), (**B**) Plasma concentration of monocyte chemoattractant protein-1 (MCP-1), (**C**) Plasma concentration of tumor necrosis factor alpha (TNFα) measured by ELISA. Data are presented as means ± S.E.M. Statistical analysis was performed by *t*-test (n=6–8).

We did not observe elevated levels of plasma C-reactive protein (CRP – an acute inflammation marker) or monocyte chemoattractant protein-1 (MCP-1) indicating no chronic systemic inflammation between WKY rats and SHRSP ones (Fig. 2A, B). The absence of peripheral inflammation was further confirmed by the measurement of unchanged levels of pro-inflammatory cytokines in plasma (TNFα and interleukin-6 (IL-6) – undetectable) (Fig. 2C).

### Signs of oxidative stress in the kidney and heart

**Fig. 3:**
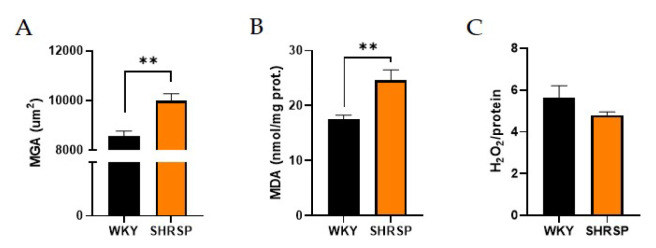
Kidney histology: (**A**) Mean glomerular area, (**B**) Lipid peroxidation measured by TBARS method in the kidney, (**C**) H_2_O_2_ production measured by Amplex Red Hydrogen Peroxide Assay Kit in the kidney. Data are presented as means ± S.E.M. Statistical analysis was performed by *t*-test. Significance is ** p<0.01 WKY vs. SHRSP (n=6–8). MGA, mean glomerular area; MDA, malondialdehyde; H_2_O_2_, hydrogen peroxide; TBARS, thiobarbituric acid-reactive substances.

Renal morphological changes were confirmed histologically. SHRSP rats had significantly larger mean glomerular area (MGA) compared to the WKY control group (Fig. 3A). On the other hand, renal damage, as determined by the glomerulosclerosis index (GSI) and tubulointerstitial injury (TII), was not observed between these two experimental groups. Similarly, plasma urea concentration remained at physiological levels in both groups, indicating no evidence of kidney dysfunction (Table 2). The only significant change in renal parenchyma was significantly increased levels of malondialdehyde (MDA) measured by thiobarbituric acid-reactive substances (TBARS) method confirming increased lipid peroxidation in SHRSP rats (Fig. 3B). However, this was not accompanied by a higher production of H_2_O_2_, as measured by the Amplex Red Assay (Fig. 3C).

**Fig. 4:**
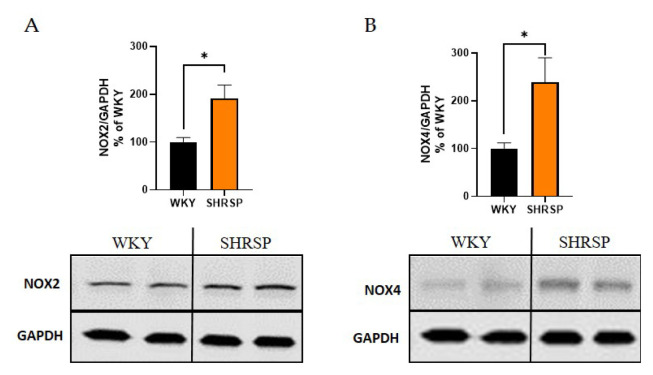
Markers of oxidative stress measured by Western blot in the heart tissue. (**A**) Quantification of the protein expression of NOX2 and (**B**) NOX4. Data are presented as means ± S.E.M. Statistical analysis was performed by *t*-test. Significance is * p<0.05 SHRSP vs. WKY (n=5–6). NOX2, NADPH oxidase 2; NOX4, NADPH oxidase 4; GAPDH, glyceraldehyde 3-phosphate dehydrogenase.

Increased oxidative stress was also detected in the heart tissue of SHRSP rats by Western blot analysis. The expression of the NADPH oxidase 2 (NOX2) and NADPH oxidase 4 (NOX4) proteins were significantly higher in SHRSP rats compared to WKY rats (Fig. 4A, B).

### SHRSP rats had mild glucose intolerance

**Fig. 5:**
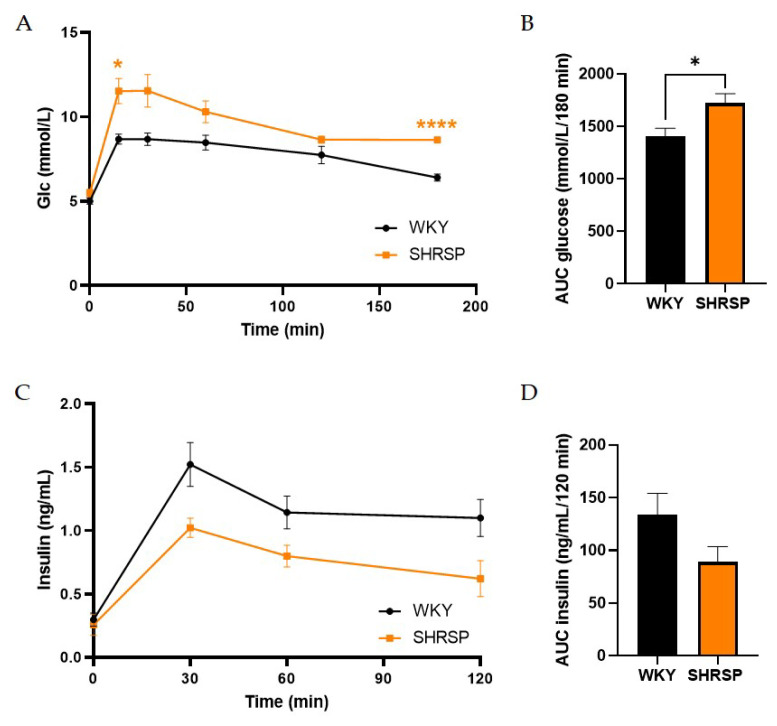
Data from oral glucose tolerance test (OGTT): (**A**) A mild glucose intolerance in SHRSP rats, (**B**) Corresponding AUC of glucose, (**C**) Plasma levels of insulin during OGTT and (**D**) Corresponding AUC of insulin in SHRSP rats and their controls WKY. Data are presented as means ± S.E.M. Statistical analysis was performed by two-way ANOVA with Sidak’s multiple comparisons test or by *t*-test. Significance is * p<0.05, **** p<0.0001 SHRSP vs. WKY (n=7–8).

Although the levels of fasted glycemia and insulinemia did not differ between both genotypes (Table 3), the results from oral glucose tolerance test (OGTT) showed mild glucose intolerance in SHRSP rats compared to WKY ones, especially at the final measurement of glycemia at the time point 180 min, where it did not decrease to baseline values as in the controls (Fig. 5A). This is also reflected in the significantly increased the glucose area under the curve (AUC) (Fig. 5B). Slightly higher insulin levels during OGTT in WKY animals could keep glucose tolerance normal as is demonstrated also by AUC (Fig. 5C, D).

### The NMR-based metabolomics in urine

**Fig. 6:**
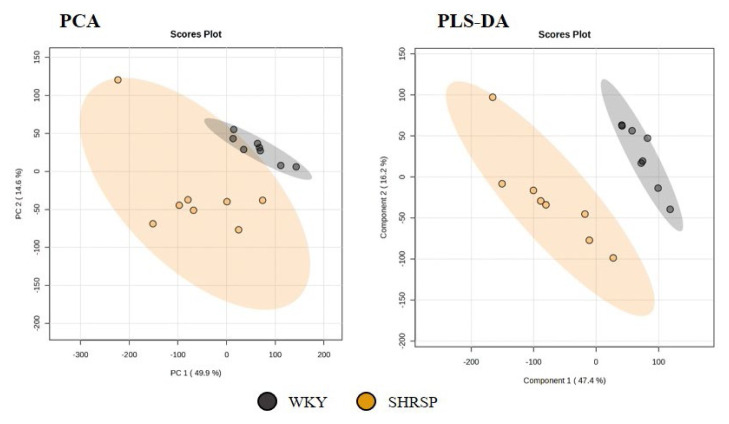
Score plots of PCA and PLS-DA models of WKY and SHRSP rats’ urine. The leave-out-one cross-validation results for 3 principal components: accuracy=1.00, R2=0.98, Q2=0.84 (n=8).

NMR metabolomic analysis was based on the comparison of the urinary metabolome of the SHRSP strain and normotensive WKY controls, both by a non-targeted multivariate-based approach and by targeted univariate analysis. No outlier was detected and a trend in group separation was observed using PCA, with the SHRSP group showing significantly higher variability than the WKY rats. The following PLS-DA model well separated the SHRSP and WKY groups (Fig. 6). The VIP score values revealed that the difference between both strains is based mainly on the decrease in citrate, hippurate, 2-oxoglutarate, and creatinine levels along with an increase in taurine, allantoin, and betaine. However, because a large signal overlap and “positional noise” is observed in the urine spectra, the individual bins may not exactly match the assigned signals, and the described metabolite changes should be verified by further statistical approaches.

In the next step, a set of 71 well-quantifiable signals was selected in the urine spectra and subsequently used to quantify metabolic alterations. Data were calculated as percentage changes in the normalized intensities of individual signals. The results for significantly changed metabolites are summarized in Table 4.

A set of statistically significant changes was observed between the urinary profile of SHRSP and WKY rats. Tricarboxylic acid cycle metabolites (except for succinate), 1-methylnicotinamide, pyruvate and host-gut microbial co-metabolites (p-cresylglucuronide, hippurate and phenylacetylglycine) decreased considerably. On the other hand, increased levels were detected for choline and its metabolites (betaine, methylamine, and dimethylamine), glucose, acetate, orotate, taurine, alanine, allantoin, pseudouridine, and 2-hydroxyisobutyrate.

### Brain biochemistry, histology and immunohistochemistry

**Fig. 7:**
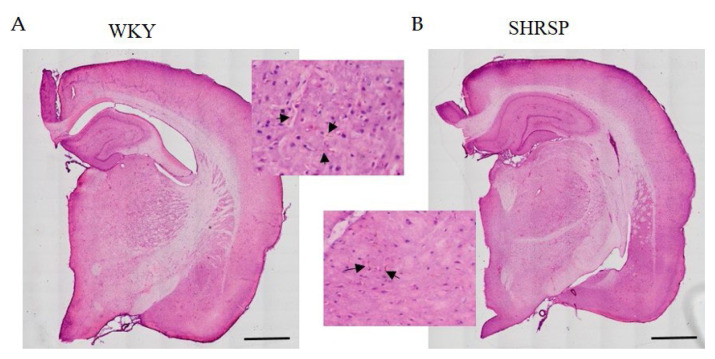
Hematoxylin-eosin staining of the whole brain slices of (**A**) WKY and (**B**) SHRSP rats showing the same manifestation of small vessel disease – accumulation of erythrocytes in capillaries (black arrows) and enlarged perivascular spaces. Scale bar = 1000 μm.

**Fig. 8:**
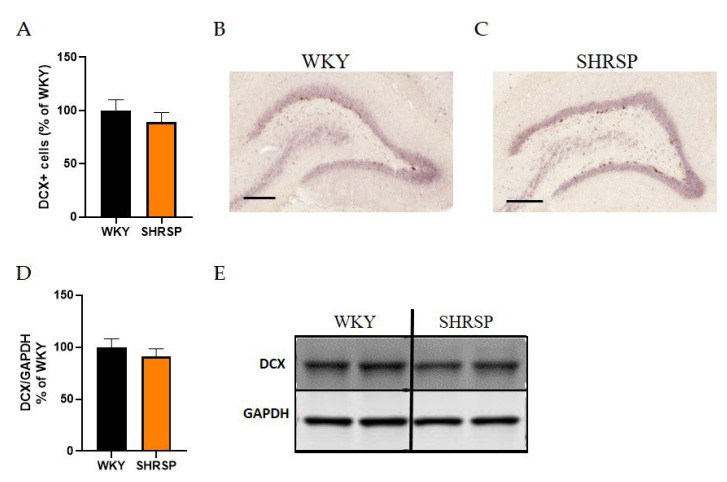
Evaluation of neurogenesis in the hippocampi of either WKY or SHRSP rats. (**A**) The percentage of doublecortin (DCX^+^) positive cells related to WKY group resulted from IHC staining, (**B**) Representative photomicrographs of the DCX^+^ cells in the hippocampus of WKY and (**C**) SHRSP rats, (**D**) Quantification of DCX protein expression in hippocampi measured by Western blot, (**E**) The Western blot image (n=5). Scale bare = 100 μm. Data are presented as means ± S.E.M. Statistical analysis was performed by *t*-test. DCX, doublecortin.

**Fig. 9:**
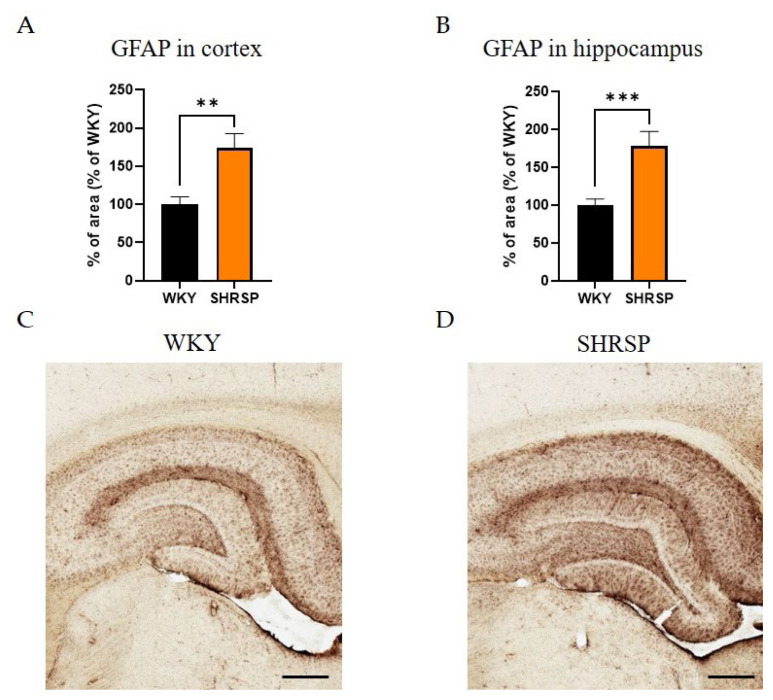
Astrocytosis in the cortex and hippocampus of SHRSP rats. (**A**) Quantification of immunohistochemical staining of GFAP in cortex and (**B**) in hippocampus, (**C**) Representative image of the hippocampus of WKY control animal and (**D**) SHRSP animal stained for the astrocyte marker GFAP. Data are presented as means ± S.E.M. Statistical analysis was performed by *t*-test. Significance is ** p<0.01, *** p<0.001 SHRSP vs. WKY (n=5). Scale bar = 250 μm. GFAP, glial fibrillary acidic protein.

Histological staining with hematoxylin-eosin (HE) was used to detect accumulated erythrocytes in small brain vessels and to visualize microbleedings. Surprisingly, the frequency of small vessel disease manifestations in SHRSP rats was the same as in the WKY controls (Fig. 7). Immunostaining for doublecortin (DCX), a marker for newly generated neurons (DCX^+^) in the dentate gyrus of the hippocampus region, did not show significant changes in the number of doublecortin-positive cells between experimental groups (Fig. 8). This result was confirmed by Western blot measurement. The photomicrographs of immunohistochemically stained brain sections in Figure 9 show that SHRSP rats have up-regulated reactivity of astrocytic marker glial fibrillary acidic protein (GFAP) compared to WKY controls in both the cortex and hippocampus. Regarding collagen deposition in cerebral arteries, we did not observe any changes in the content of type IV collagen (Col IV) in the microvessels of both SHRSP and WKY rats (data not shown).

**Fig. 10:**
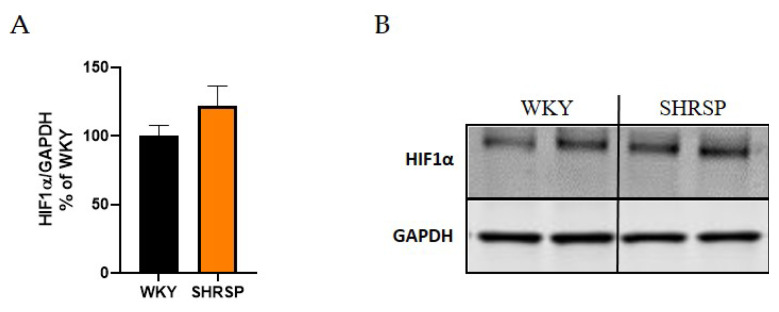
Western blot analysis of the expression of HIF1α protein in the hippocampus. (**A**) The graph showing analyzed data of HIF1α expression in hippocampi of WKY and SHRSP rats, (**B**) The Western blot image of HIF1α protein (n=5). Data are presented as means ± S.E.M. Statistical analysis was performed by *t*-test. HIF1α, hypoxia-inducible factor-1α.

We also investigated the protein expression of hypoxia-inducible factor-1α (HIF1α) in hippocampi of 32-week-old SHRSP and age-matched WKY rats. As shown in Figure 10, no significant differences were observed between SHRSP and WKY rats in the studied brain region. Additionally, we assessed the protein levels of aquaporin-4 (AQP4) in the cerebral cortex and hippocampus of SHRSP and WKY rats, and again, no significant differences were found between the two experimental groups (data not shown).

## Discussion

Our study investigated the relationship between metabolic, cardiovascular, and brain comorbidities in 32-week-old SHRSP. Given the animal’s age, which is considered critical for this strain, we anticipated to observe changes in small cerebral vessels that could be linked to metabolic and cardiovascular alterations. However, we found no such changes, even at this supposedly critical age.

To comprehensively characterize this experimental model, we included an analysis of metabolic parameters. Significantly lower levels of total cholesterol in SHRSP rats are generally well-documented when compared to WKY rats. However, less attention has been given to adipokines, particularly adiponectin and leptin. Decreased plasma leptin levels in SHRSP rats may reflect the lower body weight of these animals, but could also result from pronounced lipoatrophy [22]. Due to a standard diet without any supplementation, SHRSP animals in our study developed only mild glucose intolerance, which was not accompanied by insulin resistance. The well-established diabetic model induced by streptozotocin administration in SHRSP animals is often used to study diabetes associated with hypertension [23].

Earlier research demonstrated that SHRSP rats develop hypertension accompanied by cardiac hypertrophy and kidney enlargement [24–26]. Although we did not measure blood pressure in this study, the observed cardiac and renal hypertrophy in SHRSP rats suggests elevated blood pressure compared to WKY controls. Renal hypertrophy points to potential end-organ damage. Castiglioni *et al.* proposed that salt-loaded SHRSP rats could serve as a model for studying cardiac hypertrophy and nephropathy [27].

Oxidative stress has been implicated in the pathophysiology of hypertension and related diseases [28,29]. SHRSP rats have been shown to exhibit increased vascular superoxide release and reduced total plasma antioxidant capacity [30]. The primary source of reactive oxygen species (ROS) is NADPH oxidase, with NOX2 being the main contributor to ROS production in phagocytic cells. In our study, we examined oxidative stress in peripheral tissues (heart, kidney) and found significantly elevated expressions of NOX2 and NOX4 proteins in the hearts of SHRSP rats. These findings align with those of Umemoto *et al.* [31], who reported that 18-week-old SHRSP animals exhibited a 3.5 fold higher protein expression of the NOX2 marker p22phox compared to WKY rats, along with increased NADPH oxidase activity in heart tissue. Slightly different results are obtained when analyzing Nox mRNA levels. The aortic media of 32-week-old SHRSP rats displayed higher levels of Nox1 and Nox4 mRNA, while mRNA expressions of gp91phox and p22phox (both NOX2 markers) were comparable between SHRSP and WKY rats [32]. One limitation of our study is the absence of oxidative stress analysis in the brain. However, based on recent findings, it can be reasonably assumed that hypertensive SHRSP rats would display upregulated NOX levels in various brain regions [33,34].

Additionally, earlier research on salt-loaded SHRSP rats demonstrated multiple renal injuries, including inflammation and oxidative stress [35]. Previous studies consistently demonstrate that SHRSP rats exhibit elevated levels of plasma or serum TBARS (a marker of lipid peroxidation) compared to WKY rats. Similarly, in our study, we observed increased TBARS levels in the renal parenchyma of SHRSP rats.

NMR-based metabolomics in urine aimed to identify a set of markers associated with metabolic, cardiovascular, and brain comorbidities in 32-week-old SHRSP rats by comparing their urinary profiles with WKY controls. Monitoring these comorbidities at the level of metabolic changes in urine may aid in their early non-invasive diagnosis in the future. This approach revealed a set of metabolites with significantly different concentrations in SHRSP and WKY rats. Hippurate, *p*-cresylglucuronide, and phenylacetylglycine are products of gut microbial metabolism of phenolic compounds, tyrosine, and phenylalanine, respectively. Significant differences in gut microbial community between SHRSP and WKY rats were identified by Shi and coworkers [36]. Yang *et al.* presented that gut dysbiosis is linked to hypertension in SHR rats [37]. Decreased urinary phenylacetylglycine and *p*-cresylglucuronide levels in SHR rats compared to WKY controls were described in two studies [38,39]. In a human study, Brial *et al.* showed that urine hippurate is positively associated with microbial gene richness and can be used as a marker of metabolic health [40]. 2-Hydroxyisobutyrate is a product of microbial degradation of dietary proteins. Its increased concentration in the urine of obese people was associated with reduced bacterial diversity [41]. Methylamine and dimethylamine are produced by intestinal metabolism from dietary choline; therefore, their elevated levels also reflect differences in microbiome composition. The raised concentration of methylamine in SHR compared to WKY rats was previously observed in a study by Čermáková and coworkers [39]. Decreased levels of *p*-cresylglucuronide, hippurate, and phenylacetylglycine with the increase of 2-hydroxyisobutyrate, methylamine, and dimethylamine, observed in the present study, are consistent with previously published findings and indicate a significant difference in gut microbial colonization between SHRSP and WKY rats. Betaine, formed from choline via the mitochondrial pathway, is an osmolyte protecting cells against electrolyte imbalance and osmotic stress, particularly in the kidney. Mogilnicka and coworkers reported significantly lower concentrations of betaine in blood serum, the lungs, liver, and renal medulla in SHRs compared to normotensive rats. These changes were associated with higher urinary excretion of betaine in SHRs [42]. Increased renal excretion of betaine contributes to decreased concentration of the protective osmolyte in tissues of hypertensive rats. Significantly decreased urinary concentration of 1-methylnicotinamide, observed in SHRSP rats, was analogously reported in hypertensive patients compared to the normotensive group [43]. 1-Methylnicotinamide exerts antithrombotic and anti-inflammatory effect and has the potential to reverse the impairment of nitric oxide-dependent endothelial dysfunction in aorta of diabetic or hypertriglyceridemic rats [44]. The SHRSP rats in our study have significantly raised levels of orotic acid compared to the WKY group. Choi *et al.* reported that orotic acid induces endothelial dysfunction and leads to the development of hypertension [45]. The attenuated levels of citrate, 2-oxoglutarate, and fumarate may indicate a down-regulated TCA cycle, which can be caused by an insignificant decrease of the TCA substrate pyruvate in the urine of SHRSP rats detected in the present study. The difference can also be explained by different energy expenditure and utilization in SHRSP and WKY rats. Akira and coworkers revealed citrate and 2-oxoglutarate as the most significant metabolites distinguishing between the SHR and WKY strains in young rats [38]. It was demonstrated in the human study that metabolic acidosis also may be associated with hypertension and decreases urinary citrate levels [46]. On the other hand, succinate, another metabolite of the TCA cycle, was significantly increased in SHRSP rats. Succinate is an activator of the renin-angiotensin system, a pivotal mechanism in renal diseases. The succinate accumulation in the urine may be indicative of kidney tissue damage of several origin including hypertension or metabolic diseases such as diabetes mellitus, metabolic syndrome, *etc.* [47]. Therefore, succinate has emerged as a promising non-invasive biomarker of chronic kidney disease [48]. Thus, the elevated succinate level in our model may indicate early renal injury and the mild glucose intolerance detected in SHRSP rats. Allantoin, a product of uric acid oxidation, serves as an oxidative stress biomarker in human studies [49]. In other mammals, however, it can be formed not only by free radical oxidation as in humans, but also by enzymatic oxidation. Elevated urinary allantoin is associated with increased glomerular filtration rate (GFR) when allantoin is not reabsorbed across the proximal tubule. Based on the study of SHR and Wistar rats, Chen and coworkers suggest that allantoin may act as a central antihypertensive agent [50], so its increased urinary excretion observed in our study may be related to hypertension in the SHRSP strain. Urinary pseudouridine has been discussed as a potential marker of GFR in the study of diabetic nephropathy progression in 2670 individuals with type 1 diabetes [51]. Increased alanine was observed in rat urine after inducing damage to the proximal tubule [52].

SHRSP rats were selected as a useful model of human hypertensive encephalopathy [1]. These brain changes are manifested without any artificial treatment although higher salt intake can worsen these symptoms [53]. As can be seen from our results, hematoxylin-eosin staining of the whole brain slices have shown the same accumulation of erythrocytes in both WKY and SHRSP rats and no microbleeds in any brain region of SHRSP animals were observed. This is surprising because frequency of small vessel disease should be higher in SHRSP rats. SHRSP animals were reported to exhibit microbleeds emerging at 3 months, which increase in severity and number with age [54]. In the study of Stanisavljevic *et al.* [55], SHRSP rats showed only a few microbleeds in all brain regions (cortex, hippocampus and thalamus) and no small vessel occlusion at the age of 10 months. The possible explanation could be that infarcts occurring in SHRSP rats are associated with blood-brain barrier compromise rather than vessel occlusions [56]. Studies of SHRSP rats have shown that remodeling of cerebral arterioles occur in older (6–10 month) rats and is characterized by thickening of the blood vessel wall and decrease of lumen diameter [57,58]. To induce severe cerebral injury it may be necessary to expose SHRSP to a high salt diet, which we did not do in this study.

Astrocytes are the primary intermediary cells linking blood vessels and neurons [59,60]. They provide neuronal nutrition, maintain ionic, neurotransmitter, and metabolic homeostasis, and contribute to neuroimmune functions [61,62]. GFAP is a frequently used astrocyte-specific marker protein [63], and its upregulation plays an active role in the pathological processes of many neurological diseases associated with inflammation [64,65]. Although SHRSP rats are an inbred strain, studies analyzing GFAP expression in specific brain regions often report conflicting results. While the study by Ritz *et al.* [66] showed GFAP immunostaining in the cortex and putamen of 9-month-old SHRSP rats similar to that of age-matched WKY rats, other studies suggest that GFAP expression in the cortex and hippocampus of SHRSP rats begins to increase around 20 weeks of age, and in SHR rats from approximately 35 weeks of age [33,67,68]. The increased GFAP expression observed in the cortex and hippocampus of SHRSP animals in our present study aligns with these previous findings and may represent reactive astrocytes attempting to counteract oxidative stress conditions.

Cerebral edema is a critical initial event in most types of cerebral injury, including stroke and CSVD. Several transporters, e.g. aquaporins, play important roles in facilitating water flux through the plasma membrane of many cell types [69]. AQP4 is predominantly localized on the astrocyte foot processes of glial membranes, which are in direct contact with brain capillaries [70]. AQP4 in cerebral microglia may contribute to the pathogenesis of cerebral edema, as AQP4 knockout mice exhibit significantly better survival rates than wild-type mice in a model of brain edema caused by acute water intoxication induced by focal brain ischemia [71]. In the present study, the protein expression levels of AQP4 in the cerebral cortex and hippocampus of SHRSP rats were comparable to those in the control animals. This finding suggests the absence of brain edema in these experimental animals. These results are not consistent with the study by Takemori *et al.* [72], which reported increased AQP4 expression in the cerebral cortex of 18-week-old SHRSP animals. A possible explanation for this discrepancy could be the source of Takemori’s SHRSP rats, which were purchased from Kyoto, Japan and were likely more closely related to Okamoto‘s original strain.

Hypertension could potentially lead to vascular alterations, resulting in hypoperfusion-induced hypoxia. HIF1α can also be upregulated by inflammatory signals, with oxidative stress playing a crucial role [73]. Zhang *et al.* demonstrated increased HIF1α expression in 20-week-old SHRSP rats compared to WKY rats in the frontal cortex, corpus callosum, and striatum regions [33]. However, our measurements revealed unchanged expression of HIF1α in the hippocampi of SHRSP rats.

On the other hand, our findings regarding similar levels of the membrane protein collagen IV in normotensive WKY and hypertensive SHRSP rats align with the Zhang’s observations [33]. They also reported unchanged collagen IV expression in cortical tissues, measured by both WB and immunohistochemistry. This is noteworthy, given that hypertension is known to induce remodelling of small vessel basement membranes, leading to excessive deposition of collagens and fibronectin, resulting in fibrotic changes [74,75]. Based on this and other studies [33], it seems that hypertension alone may not suffice to induce basement membrane changes within a 32-week timeframe. A longer duration of hypertension exposure might be necessary to cause such alterations.

Findings from our study indicate that SHRSP rats at 32 weeks exhibit cardiac and renal hypertrophy, likely secondary to hypertension, with associated metabolic changes and oxidative stress. Although these rats do not have gross cerebral pathology or stroke, they have indicators of neuroinflammation and astrocyte hyperreactivity. This result contrasts with earlier studies by Okamoto [1] and Sansawa *et al.* [76], which reported a 60 % survival rate at 21 weeks and 0 % at 25 weeks. Even worse outcomes have been documented when SHRSP rats were given 1 % salt in water starting at 40 days old, leading to severe hypertension and a 0 % survival rate by 90 days [77]. The reasons for these discrepancies remain unclear. Our previous study with Zucker fa/fa rats [78] demonstrated that results can vary significantly depending on the source of the animals, raising the possibility that this variability could also apply to SHRSP rats. This subject will require a further investigation.

In conclusion, we can state that we did not observe any changes in the occurrence of vascular problems in the brain, which contradicts the results of a number of other studies. This could be related to the fact that SHRSP rats from different breeders are used for the study and these animals differ significantly from the original SHRSP rats bred by Okamoto *et al.* [1]. Our results suggested, that neuroinflammation and/or oxidative stress could be a denominators of the relation among metabolic, cardiovascular and central disturbances. NMR metabolomic analysis of urine showed several significant differences between the SHRSP and WKY groups, which are consistent with previously published data for this model and can be attributed to the altered gut microbiome, hypertension, and possible renal impairment in the SHRSP strain.

## Figures and Tables

**Table 1:** Morphometric and metabolic parameters of WKY and SHRSP rats analyzed at 32 weeks of age in fasted plasma.

Group	WKY	SHRSP
*BW (g)*	399.4±7.65	356.6±5.15***
*Body temperature (°C)*	36.0±0.10	36.3±0.26
*FFA (mmol/l)*	0.98±0.16	1.03±0.09
*TG (mmol/l)*	0.83±0.12	0.73±0.08
*CHOL (mmol/l)*	2.47±0.07	1.59±0.08***
*Leptin (ng/ml)*	5.09±0.46	3.52±0.56**
*Heart/BW ratio*	0.326±0.018	0.403±0.011***
*Kidney/BW ratio*	0.610±0.007	0.847±0.015****

**Table 2:** Kidney histological analysis and plasmatic urea.

Group	WKY	SHRSP
*GSI*	0.063±0.058	0.057±0.040
*TII*	0.046±0.049	0.219±0.140
*Plasma urea (mmol/l)*	4.55±0.54	4.72±0.65

Data are presented as means ± S.E.M. Statistical analysis was performed by *t*-test (n=7–8). GSI, glomerulosclerosis index; TII, tubulointerstitial injury.

**Table 3:** Fasted glycemia and insulinemia.

Group	WKY	SHRSP
*Glucose (mmol/l)*	5.0±0.20	5.5±0.28
*Insulin (ng/ml)*	0.30±0.06	0.26±0.09

**Table 4:** Significantly changed metabolites in the urine of WKY and SHRSP groups.

Metabolite	Δ%	p-value
*1-Methylnicotinamide*	−30.54	**
*2-Oxoglutarate*	−35.35	*
*2-Hydroxyisobutyrate*	17.52	**
*Acetate*	223.62	*
*Alanine*	17.10	**
*Allantoin*	31.58	*
*Betaine*	25.02	**
*Citrate*	−34.44	*
*Dimethylamine*	13.49	*
*Fumarate*	−56.58	***
*Hippurate*	29.74	*
*Methylamine*	53.79	**
*Orotate*	52.52	***
*p-Cresylglucuronide*	−44.99	***
*Phenylacetylglycine*	−18.85	#
*Pseudouridine*	17.69	**
*Pyruvate*	−14.50	**
*Succinate*	37.77	*
*Taurine*	65.30	*

The results are expressed as the percentage change of normalized concentrations in SHRSP vs. WKY group (n=8). The statistical significance was analyzed by *t*-test.

* p<0.05,

** p<0.01,

*** p<0.001,

# – trend with p<0.1.

## Abbreviations

AQP4: Aquaporin-4
AUC: Area under the curve
BW: Body weight
CHOL: Total cholesterol
Col IV: Collagen type IV
CRP: C-reactive protein
CSVD: Cerebral small vessel disease
DCX: Doublecortin
EGTA: Egtazic acid
FFA: Free fatty acids
GAPDH: Glyceraldehyde 3-phosphate dehydrogenase
GFAP: Glial fibrillary acidic protein
GFR: Glomerular filtration rate
GSI: Glomerulosclerosis index
H_2_O_2_: Hydrogen peroxide
HE: Hematoxylin-eosin staining
HIF1α: Hypoxia-inducible factor-1α
IHC: Immunohistochemistry
IL-6: Interleukin-6
MCP-1: Monocyte chemoattractant protein-1
MDA: Malondialdehyde
MGA: Mean glomerular area
NADPH: Reduced nicotinamide adenine dinucleotide phosphate
NOX1-4: NADPH oxidase 1, 2, 3, 4
OGTT: Oral glucose tolerance test
PFA: Paraformaldehyde
PMSF: Phenylmethylsulfonyl fluoride
ROS: Reactive oxygen species
SHRSP: Stroke-prone spontaneously hypertensive rats
TBARS: Thiobarbituric acid-reactive substances
TCA cycle: Tricarboxylic acid cycle
TG: Triglycerides
TII: Tubulointerstitial injury
TNFα: Tumor necrosis factor alpha
WKY: Wistar-Kyoto rats
